# Development and validation of a prognostic nomogram to predict overall survival and cancer-specific survival for patients with anaplastic thyroid carcinoma

**DOI:** 10.7717/peerj.9173

**Published:** 2020-05-21

**Authors:** Weiwei Gui, Weifen Zhu, Weina Lu, Chengxin Shang, Fenping Zheng, Xihua Lin, Hong Li

**Affiliations:** Department of Endocrinology, the Affiliated Sir Run Run Shaw Hospital, School of Medicine, Zhejiang University, Hangzhou, Zhejiang, China

**Keywords:** Anaplastic thyroid carcinoma, Nomogram, Overall survival, Cancer-specific survival, SEER

## Abstract

**Background:**

Anaplastic thyroid carcinoma (ATC) is a rare malignant tumor with a poor prognosis. However, there is no useful clinical prognostic predictive tool for ATC so far. Our study identified risk factors for survival of ATC and created a reliable nomogram to predict overall survival (OS) and cancer-specific survival (CSS) of patients with ATC.

**Methods:**

A total of 1,404 cases of ATC diagnosed between 1983 and 2013 were extracted from on the Surveillance, Epidemiology and End Results database based on our inclusion criteria. OS and CSS were compared among patients between each variable by Kaplan–Meier methods. The Cox proportional hazards model was used to evaluate multiple prognostic factors and obtain independent predictors. All independent risk factors were included to build nomograms, whose accuracy and practicability were tested by concordance index (C-index), calibration curves, ROC curves, DCA, net reclassification improvement (NRI) and integrated discrimination improvement (IDI).

**Results:**

Historic stage, tumor size, surgery and radiotherapy were independent risk factors associated with ATC according to multivariate Cox regression analysis of OS. However, gender was also an important prognostic predictor in CSS besides the factors mentioned above. These characteristics were included in the nomograms predicting OS and CSS of patients with ATC. The nomograms predicting OS and CSS performed well with a C-index of 0.765 and 0.773. ROC curves, DCA, NRI and IDI suggested that the nomogram was superior to TNM staging and age.

**Conclusion:**

The proposed nomogram is a reliable tool based on the prediction of OS and CSS for patients with ATC. Such a predictive tool can help to predict the survival of the patients.

## Introduction

Anaplastic thyroid carcinoma (ATC) is a rare but aggressive thyroid carcinoma in humans with an estimated incidence of 1–2 cases per million people in the USA. It comprises only 1–2% of all thyroid cancers and accounts for 3.6% of all human cancers (Davies & Welch, 2006; Siegel, Miller & Jemal, 2016; Gilliland et al., 1997). Unlike well-differentiated thyroid carcinoma diagnosed by fine-needle aspiration, ATC is diagnosed by clinical manifestations including rapidly growing thyroid mass, dyspnea and so on (Haddad et al., 2015). Half of the patients exhibited distant metastasis at the time of diagnosis (Glaser et al., 2016). ATC is almost lethal with the median OS of the patients from the time of diagnosis reported to be 3–5 months (Kebebew et al., 2005). Most death of ATC patients is attributable to rapid progression of tumor which often results in suffocation (Sugitani et al., 2012). Significant independent predictors reported to be associated with poorer survival in ATC are advanced stage, older age, distant metastases, large primary tumor size and elevated white blood count, while better prognosis has been observed when ATC coexists with differentiated thyroid carcinoma (Orita et al., 2011; Perros et al., 2014; Kitamura et al., 1999). Current treatments for ATC include surgery, chemotherapy and radiotherapy, and the optional therapy tailored to each patient depends on their own risk estimation (Giuffrida & Gharib, 2000). Considering the aggressive behavior of this disease, prompt assessments of the patients are desirable in order to make a specific management plan.

Because of the need of precise risk evaluation of ATC patients and the lack of a specific and practical predictive tool, establishing a predictive model that incorporates numerous predictors related to the probability of OS and CSS becomes desirable. Among all the available models, a nomogram can provide an evidence-based, individualized, highly precise risk evaluation (Yang, Shen & Sakamoto, 2013). Nomograms are widely used among several kinds of diseases, such as gallbladder cancer (Xiao et al., 2019), Crohn’s disease (Zhu et al., 2020), non-small cell lung cancer (Zhang et al., 2019) and so on. Therefore, the purpose of this study is to identify the most relevant prognosticators and combine them into nomograms to better define the risk factors of an individual patient.

## Methods

### Study population

**Figure 1:**
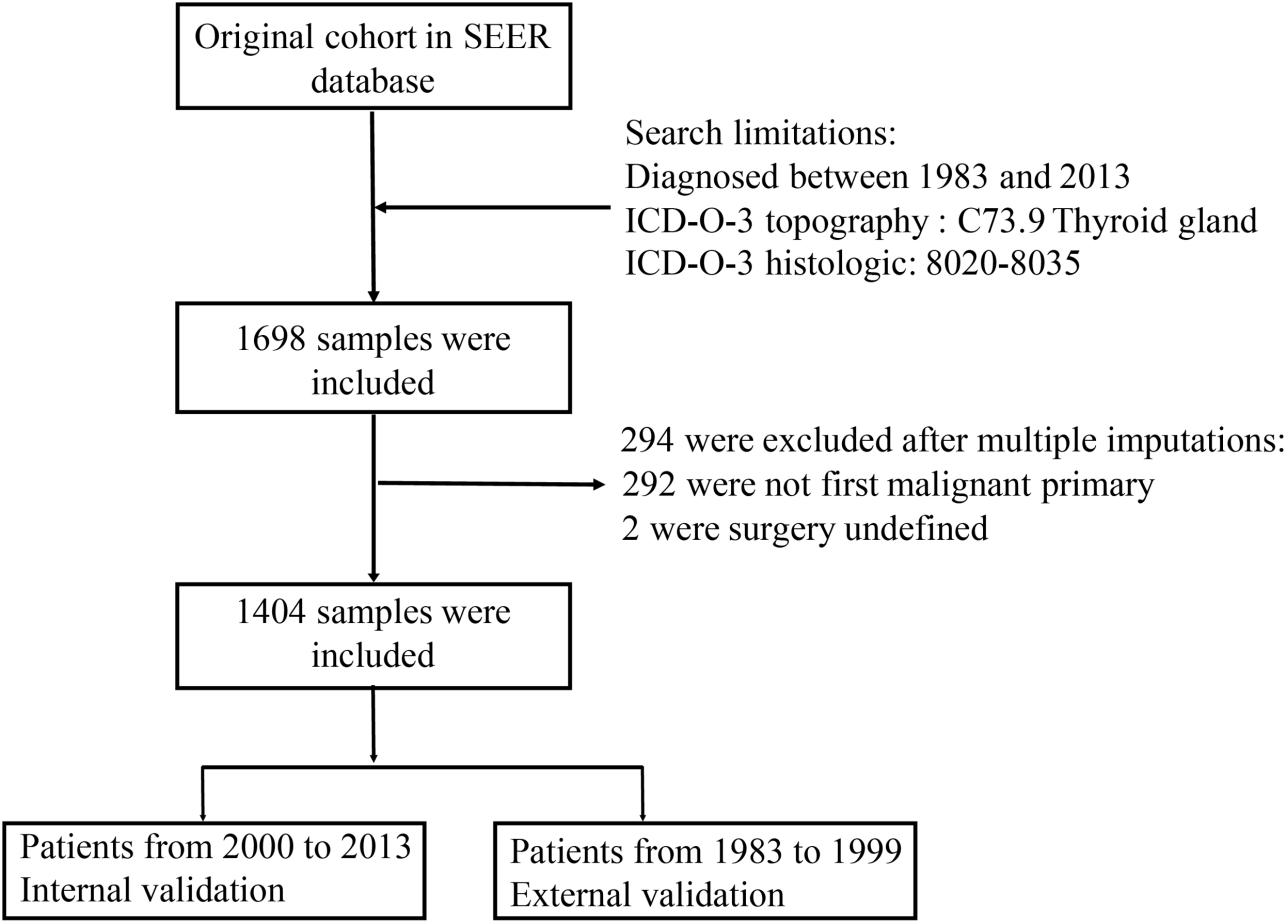
Flow chart for selection the data from SEER database.

Cases diagnosed as anaplastic thyroid cancer during 1983–2013 were extracted from the Surveillance, Epidemiology and End Results (SEER-18) registries (National Cancer Institute, 2015). It includes data from 18 high-quality registries, which represents about 26% of the US population. Information about demographic, tumor characteristics and treatment for each case were obtained from the medical record. We identified anaplastic thyroid cancer patients using the International Classification of Diseases for Oncology, Third Edition (ICD-O-3). Consistent with previous research (Lim et al., 2017), only patients with ICD-O-3 histologic codes of 8020–8035 and topography code of C73 were included. Missing data including patients’ unknown race, radiation treatment and survival time were imputed with multiple imputations. We carried out multiple imputations based on R version 3.5.1 using the R package of mice. Then patients were excluded if they were not first malignant primary and had undefined surgery. We divided our data into internal validation and external validation (Fig. 1).

### Covariates

The demographics of patients (age, sex, race and year of diagnosis), tumor characteristics (SEER historic stage A and tumor size) and treatment (surgery and radiotherapy) were gathered from the database. Patients were divided into <65 and ≥65 years old groups according to age at diagnosis. We have used splines to explore non-linear relationships between age at diagnosis and survival months. The result was shown as Fig. S1. As shown in the figure, survival months of patients exhibited a steep decline between 60 and 80 years old. It’s obvious that the age in this range is related to the survival months which was coherent with numerous studies. Some studies reported that age ≥70 years old was an indicator of poor survival for ATC patients (Onoda et al., 2020; Sugitani et al., 2012). Some studies reported that people younger than 60 years old had superior prognosis (Araque, Gubbi & Klubo-Gwiezdzinska, 2020; Roche et al., 2018). Taken these into consideration, we divided patients into <65 and ≥65 years old groups. SEER historic stage A was an indicator to distinguish tumor stage: localized (confined to thyroid), regional (tumor extend to adjacent tissues, blood vessels and lymph nodes), distant (distant metastasis) and unstaged. Only after 1983, SEER database began to record tumor size. So far, there were 3 codes to identify tumor size: extent of disease-4 codes (EOD-4) for 1983–1987, extent of disease-10 codes (EOD-10) for 1988–2003, and collaborative staging codes (CS) for 2004–2013. We combined these codes to define tumor size in patients with anaplastic thyroid cancer. Furthermore, patients were divided into lobectomy, total/near total thyroidectomy and no cancer-direct surgery/unknown groups according to the surgical procedure.

### Outcomes

In this study, the outcomes we were interested in were overall survival (OS) and cancer-specific survival (CSS), which were defined as the interval from diagnosis until death for all causes and the interval from diagnosis until death only due to this tumor, respectively. SEER database provides specific codes to define both outcomes.

### Data analysis

Only the first matching record was selected into analysis. The Kaplan–Meier method was used to compare OS and CSS between each covariate, and their differences were assessed by log-rank test. Multivariate Cox regression analysis was used to determine the independent risk factors of OS and CSS for anaplastic thyroid cancer patients through the SPSS statistical software (version 24). Besides, as various cause-specific deaths were competing risk events, we also used competing risk analyses as additional analyses to make our results more credible. We carried out competing risk analyses using the R package of cmprsk (Scrucca, Santucci & Aversa, 2007). All independent risk factors analyzed by multivariate Cox regression were included to construct the nomograms.

Nomograms were built and validated by R version 3.5.1, using the R package of rms and survival (Frank, 2019). We used bootstrap self-sample verification while establishing nomogram prediction model based on the data calculated by multivariate logistic regression analysis. Internal calibration and external calibration curves were used to assess the accuracy of the nomograms. To create a calibration diagram, we used Cox regression predictive model. *X*-axis means predictive survival time and *Y*-axis means actual survival time. In a perfectly predictive model, predictive rates would fall on a 45° diagonal line. We used concordance index (C-index) to describe the discrimination between predicted probability and actual outcome. The value of the C-index should fall between 0.5 and 1.0, with 0.5 presenting random chance and 1.0 presenting a perfect accordance. Generally, the value greater than 0.7 indicated a strong predictive ability. To evaluate the accuracy and practicability of our nomogram, we carried out receiver operating characteristic (ROC) analysis and Decision Curve Analysis (DCA) based on the R package of survival ROC and rmda respectively (Rousson & Zumbrunn, 2011). ROC represented sensitivity and specificity of nomogram while couldn’t ignore false positive rate and false negative rate. DCA represented net benefit of a clinical decision. To compare the accuracy of our nomogram with that of traditional TNM staging system, the net reclassification index (NRI) and integrated discrimination index (IDI) were analyzed based on the R package of nricens and predictABEL as reported (Zhou et al., 2019).

A two-sided *P* value of 0.05 was considered statistically significant. The Kaplan–Meier curves were built by GraphPad Prism 6.0.

## Results

### Patient characteristics

A total of 1,404 patients diagnosed as ATC during 1983–2013 was included in the analysis. We divided our patients into two parts: internal validation and external validation. Internal validation contained patients diagnosed from 2000 to 2013, and external validation contained patients diagnosed from 1983 to 1999. Demographic, tumor characteristics and treatment information of patients in internal validation were presented in Table 1 (Year of diagnosis of patients in external validation ranged from 1983–1999). Elder (654 (64.5%)), female (630 (62.1%)) and white patients (815 (80.4%)) constituted the main part of the cases. Median age at diagnosis of cases was 70 years old and median follow-up time was 2 months. Most patients (567 (56.1%)) underwent tumor metastasis, while only 61 (6.0%) patients had localized tumors. When stratifying by tumor size, more than half of the patients had a tumor size greater than 4 cm. In addition, of the patients who underwent surgery, 177 (17.5%) cases underwent lobectomy and 287 (28.3%) cases underwent total or near total thyroidectomy. And 571 (56.3%) patients received radiotherapy.

**Table 1:** Demographics, tumor characteristics and treatment information of 1014 patients with anaplastic thyroid carcinoma.

Characteristics	Internal validation		External validation	
	*N*	%	*N*	%
Age group				
<65	360	35.5	123	31.5
≥65	654	64.5	267	68.5
Sex				
Male	384	37.9	145	37.2
Female	630	62.1	245	62.8
Race				
White	815	80.4	321	82.3
Black	84	8.3	23	5.9
Others	115	11.3	46	11.8
Year of diagnosis				
2000–2004	311	30.7		
2005–2009	367	36.2		
2010–2013	336	33.1		
SEER historic stage A				
Localized	61	6.0	31	7.9
Regional	337	33.2	155	39.7
Distant	569	56.1	166	42.6
Unstaged	47	4.6	38	9.7
Tumor size				
≤2.0 cm	31	3.1	19	4.9
2.1–4.0 cm	115	11.3	36	9.2
>4 cm	573	56.5	162	41.5
Unknown	295	29.1	173	44.4
Surgery				
Lob	177	17.5	68	17.4
Total thy	287	28.3	84	21.5
No cancer-direct/Unknown	550	54.2	238	61.0
Radiotherapy				
No	443	43.7	133	34.1
Yes	571	56.3	257	65.9

**Note:**

Lob, Lobectomy; Thy, Thyroidectomy.

### Survival analysis

**Figure 2:**
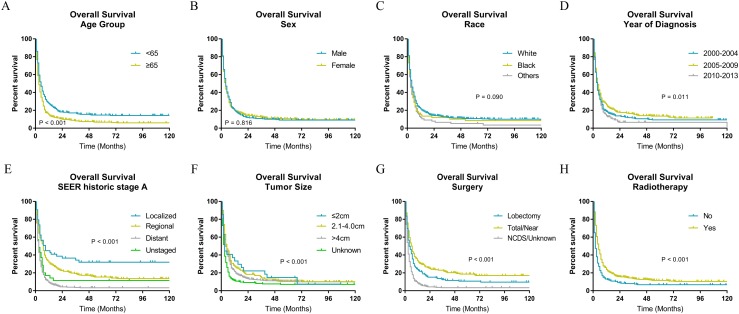
Overall survival of patients with ATC by different variables. (A) Age, (B) sex, (C) race, (D) year of diagnosis, (E) SEER historic stage A, (F) tumor size, (G) surgery and (H) radiotherapy. Total/Near, Total thyroidectomy/Near thyroidectomy; NCDS, No cancer-direct surgery.

**Figure 3:**
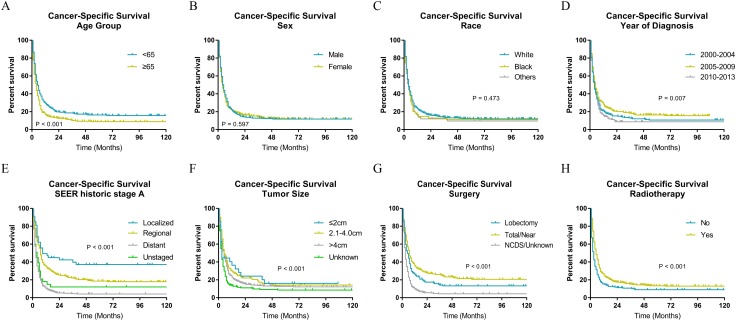
Cancer-specific survival of patients with ATC by different variables. (A) Age, (B) sex, (C) race, (D) year of diagnosis, (E) SEER historic stage A, (F) tumor size, (G) surgery and (H) radiotherapy. Total/Near, Total thyroidectomy/Near thyroidectomy; NCDS, No cancer-direct surgery.

The Kaplan–Meier analysis (Figs. 2 and 3) showed age ≥65 years old rather than age <65 years old was significantly associated with worse OS and CSS for patients with ATC. It also revealed that sex and race was not significantly associated with OS and CSS. When considering year of diagnosis, we found that patients diagnosed from 2010 to 2013 had worst survival. When stratifying by tumor stage, ATC with distant metastasis showed severely poorer OS and CSS than a localized or regional tumor. The results also indicated ATC with larger tumor size had significantly worse survival. OS and CSS were significantly better for patients who underwent total or near total thyroidectomy than those who underwent lobectomy, while patients with no cancer-direct surgery or unknown surgical procedure had the worst survival. As for radiotherapy, survival was better for patients receiving radiotherapy than patients who did not receive radiotherapy.

### Independent factors associated with OS and CSS

The Cox proportional hazards regression analysis was used to determine independent risk factors for OS (Table 2), and Cox proportional hazards and competing risk analyses were used to determine independent risk factors for CSS (Table 3). In our study, age ≥65 years old was a risk factor with worse OS (HR, 1.525; (95% CI [1.326–1.752]); *P* < 0.001). The results also indicated that tumor stage (distant vs localized: HR, 2.641; (95% CI [1.923–3.628]); *P* < 0.001); regional vs localized: HR, 1.427; (95% CI [1.031–1.974]); *P* = 0.032, unstaged vs localized: HR, 2.138; (95% CI [1.393–3.282]; *P* = 0.001), tumor size (unknown vs ≤2.0 cm: HR, 1.647; (95% CI [1.115–2.433]); *P* = 0.012), surgery (total/near total thyroidectomy vs lobectomy: HR, 0.715; (95% CI [0.583–0.878]); *P* = 0.001; no cancer-direct surgery/unknown vs lobectomy: HR, 1.702; (95% CI [1.420–2.040]); *P* < 0.001) and radiotherapy (yes vs no: HR, 0.508; (95% CI [0.445–0.580]); *P* < 0.001). Besides, sex, race and year of diagnosis were not significantly associated with survival of patients. And after adjusting for other available variables, age ≥65 years old was independently associated with worse OS (HR, 1.471; (95% CI [1.276–1.696]); *P* < 0.001) and tumor size, surgery and radiotherapy were also independent risk factors. As for CSS (Table 3), we found that age, stage, surgery and radiotherapy were risk factors according to univariate survival analysis. After adjusting for other available variables, we found that age ≥65 years old was an independent risk factor with worse CSS (HR, 1.408; (95% CI [1.216–1.631]); *P* < 0.001), which was consistent with the results of competing risk analysis (HR, 1.205; (95% CI [1.053–1.379]); *P* < 0.01). Similar to age, stage, surgery and radiotherapy were also independent risk factors according to both multivariate survival analysis and competing risk analysis. While sex, race, year of diagnosis and tumor size were not associated with prognosis of ATC patients.

**Table 2:** Cox proportional hazards regression analysis of overall survival.

Characteristics	Univariate analysis	*P*	Multivariate analysis	*P*
	HR (95% CI)		HR (95% CI)	
Age group				
<65	1.00 (reference)		1.00 (reference)	
≥65	1.525 [1.326–1.752]	<0.001	1.471 [1.276–1.696]	<0.001
Sex				
Male	1.00 (reference)		1.00 (reference)	
Female	1.065 [0.931–1.218]	0.357	1.032 [0.900–1.184]	0.651
Race				0.614
White	1.00 (reference)		1.00 (reference)	
Black	1.166 [0.921–1.474]	0.202	1.070 [0.844–1.358]	0.576
Others	1.253 [1.023–1.535]	0.029	1.097 [0.892–1.348]	0.382
Year of diagnosis				0.573
2000–2004	1.00 (reference)		1.00 (reference)	
2005–2009	0.907 [0.775–1.063]	0.229	0.932 [0.790–1.100]	0.405
2010–2013	1.044 [0.885–1.230]	0.611	1.009 [0.849–1.199]	0.922
SEER historic stage A				
Localized	1.00 (reference)		1.00 (reference)	
Regional	1.427 [1.031–1.974]	0.032	1.506 [1.086–2.088]	0.014
Distant	2.641 [1.923–3.628]	<0.001	2.285 [1.655–3.156]	<0.001
Unstaged	2.138 [1.393–3.282]	0.001	1.272 [0.816–1.982]	0.288
Tumor size				0.015
≤2.0 cm	1.00 (reference)		1.00 (reference)	
2.1–4.0 cm	0.964 [0.633–1.466]	0.863	0.848 [0.555–1.297]	0.447
>4 cm	1.144 [0.781–1.674]	0.490	0.884 [0.601–1.301]	0.532
Unknown	1.647 [1.115–2.433]	0.012	1.139 [0.760–1.705]	0.529
Surgery				<0.001
Lob	1.00 (reference)		1.00 (reference)	
Total thy	0.715 [0.583–0.878]	0.001	0.802 [0.651–0.987]	0.037
No cancer-direct/Unknown	1.702 [1.420–2.040]	<0.001	1.492 [1.231–1.807]	<0.001
Radiotherapy				
No	1.00 (reference)		1.00 (reference)	
Yes	0.508 [0.445–0.580]	<0.001	0.533 [0.465–0.611]	<0.001

**Table 3:** Cox proportional hazards regression analysis and competing risk analysis of cancer-specific survival.

Characteristics	Univariate analysis	*P*	Multivariate analysis	*P*	Competing risk analysis	*P*
	HR (95% CI)		HR (95% CI)		HR (95% CI)	
Age group						
<65	1.00 (reference)		1.00 (reference)		1.00 (reference)	
≥65	1.464 [1.268–1.691]	<0.001	1.408 [1.216–1.631]	<0.001	1.205 [1.053–1.379]	<0.01
Sex						
Male	1.00 (reference)		1.00 (reference)		1.00 (reference)	
Female	1.086 [0.944–1.248]	0.249	1.067 [0.924–1.230]	0.377	1.123 [0.979–1.289]	0.10
Race				0.812		
White	1.00 (reference)		1.00 (reference)		1.00 (reference)	
Black	1.139 [0.891–1.457]	0.299	1.051 [0.819–1.349]	0.695	1.005 [0.767–1.317]	0.97
Others	1.193 [0.963–1.479]	0.106	1.064 [0.855–1.324]	0.579	1.057 [0.840–1.330]	0.64
Year of diagnosis				0.388		
2000–2004	1.00 (reference)		1.00 (reference)		1.00 (reference)	
2005–2009	0.879 [0.745–1.036]	0.124	0.905 [0.762–1.074]	0.254	0.88 [0.744–1.042]	0.14
2010–2013	1.034 [0.872–1.225]	0.703	1.004 [0.840–1.200]	0.967	0.993 [0.838–1.176]	0.93
SEER historic stage A				<0.001		
Localized	1.00 (reference)		1.00 (reference)		1.00 (reference)	
Regional	1.549 [1.087–2.206]	0.015	1.625 [1.138–2.319]	0.008	1.606 [1.146–2.250]	<0.01
Distant	2.974 [2.105–4.202]	<0.001	2.580 [1.816–3.665]	<0.001	2.407 [1.723–3.362]	<0.01
Unstaged	2.448 (1.553 to 3.856)	<0.001	1.460 (0.913 to 2.335)	0.114	1.625 (1.035 to 2.552)	0.04
Tumor size				0.024		
≤2.0 cm	1.00 (reference)		1.00 (reference)		1.00 (reference)	
2.1–4.0 cm	1.002 [0.644–1.559]	0.993	0.879 [0.562–1.374]	0.571	1.027 [0.672–1.571]	0.90
>4 cm	1.184 [0.791–1.771]	0.411	0.902 [0.600–1.357]	0.622	0.985 [0.657–1.475]	0.94
Unknown	1.715 [1.136–2.590]	0.010	1.162 [0.759–1.778]	0.49	1.185 [0.778–1.806]	0.43
Surgery				<0.001		
Lob	1.00 (reference)		1.00 (reference)		1.00 (reference)	
Total Thy	0.705 [0.570–0.873]	0.001	0.782 (0.630 to 0.971)	0.026	0.774 (0.641 to 0.936)	<0.01
No cancer-direct/Unknown	1.703 [1.411–2.055]	<0.001	1.461 (1.198 to 1.782)	<0.001	1.252 (1.046 to 1.499)	0.01
Radiotherapy						
No	1.00 (reference)		1.00 (reference)		1.00 (reference)	
Yes	0.532 [0.463–0.611]	<0.001	0.558 [0.484–0.644]	<0.001	0.718 [0.624–0.825]	<0.01

**Note:**

Lob, Lobectomy; Thy, Thyroidectomy.

### Nomogram construction and validation

**Figure 4:**
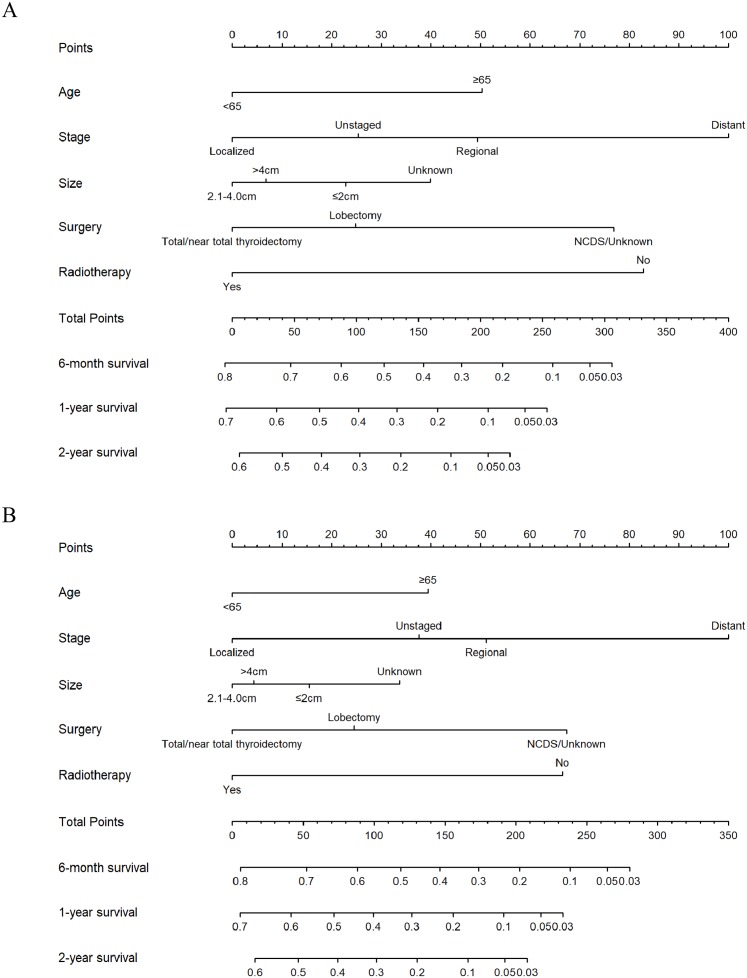
Nomograms for predicting 6-month, 1-year, 2-year OS and CSS rates of patients with ATC. (A) Predicting 6-month, 1-year, 2-year OS and (B) predicting 6-month, 1-year, 2-year CSS. NCDS, No cancer-direct surgery.

All independent risk factors derived from Cox proportional hazards regression analysis were used to construct the nomograms, which were effective tools to predict 6-month, 1-year and 2-year OS or CSS (Fig. 4). Therefore, age group, tumor stage, tumor size, surgery and radiotherapy were included in the nomogram for OS and CSS. Both for OS and CSS, tumor stage contributed most for survival. Using the nomograms to assess the prognosis of a patient requires adding the scores corresponding to each predictor to get a total score. The total score corresponds to 6-month, 1-year, 2-year overall or CSS rate.

**Figure 5:**
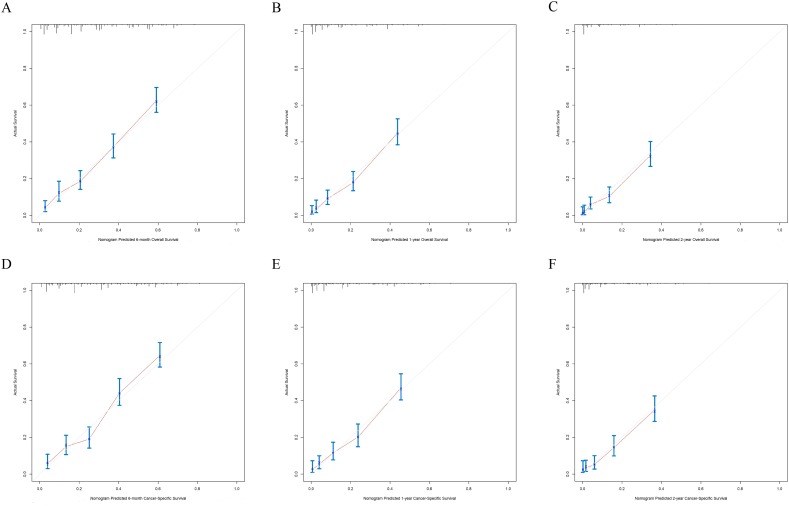
The internal calibration curves of the nomogram predicting 6-month, 1-year, 2-year OS and CSS. *X*-axis represents the nomogram predicted survival, and *Y*-axis represents the actual survival. (A) Calibration curve of nomogram predicting 6-month OS, (B) calibration curve of nomogram predicting 1-year OS, (C) calibration curve of nomogram predicting 2-year OS, (D) calibration curve of nomogram predicting 6-month CSS, (E) calibration curve of nomogram predicting 1-year CSS and (F) calibration curve of nomogram predicting 2-year CSS.

**Figure 6:**
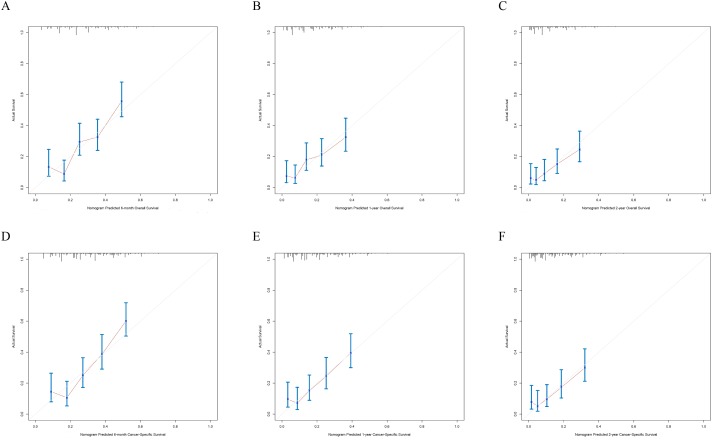
The external calibration curves of the nomogram predicting 6-month, 1-year, 2-year OS and CSS. *X*-axis represents the nomogram predicted survival, and *Y*-axis represents the actual survival. (A) Calibration curve of nomogram predicting 6-month OS, (B) calibration curve of nomogram predicting 1-year OS, (C) calibration curve of nomogram predicting 2-year OS, (D) calibration curve of nomogram predicting 6-month CSS, (E) calibration curve of nomogram predicting 1-year CSS and (F) calibration curve of nomogram predicting 2-year CSS.

The nomograms were validated internally and externally. The C-index values of internal validation were 0.765 for OS and 0.773 for CSS, respectively, indicating acceptable discriminations. In addition, the calibration curves for OS and CSS demonstrated great accordance between predicted survival by nomogram and actual observed survival. Internal calibration curves (Fig. 5) and external calibration (Fig. 6) curves suggested that our nomograms had proper calibrations.

**Figure 7:**
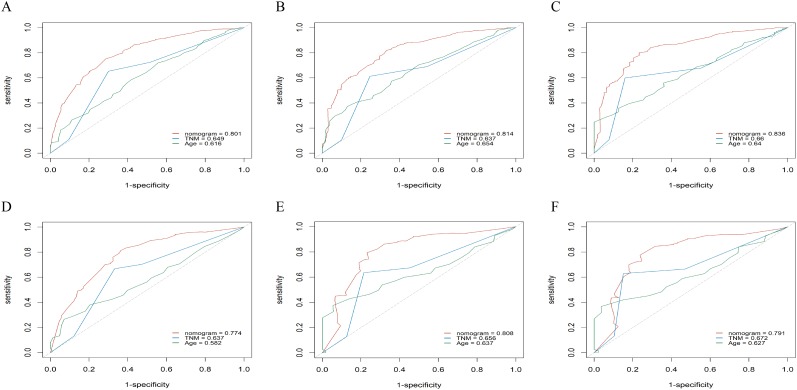
The ROC curves of the nomogram predicting 6-month, 1-year, 2-year OS and CSS. *X*-axis represents false positive rate, and *Y*-axis represents sensitivity. (A) ROC curve of the nomogram predicting 6-month OS, (B) ROC curve of the nomogram predicting 1-year OS, (C) ROC curve of the nomogram predicting 2-year OS, (D) ROC curve of the nomogram predicting 6-month CSS, (E) ROC curve of the nomogram predicting 1-year CSS and (F) ROC curve of the nomogram predicting 2-year CSS.

**Figure 8:**
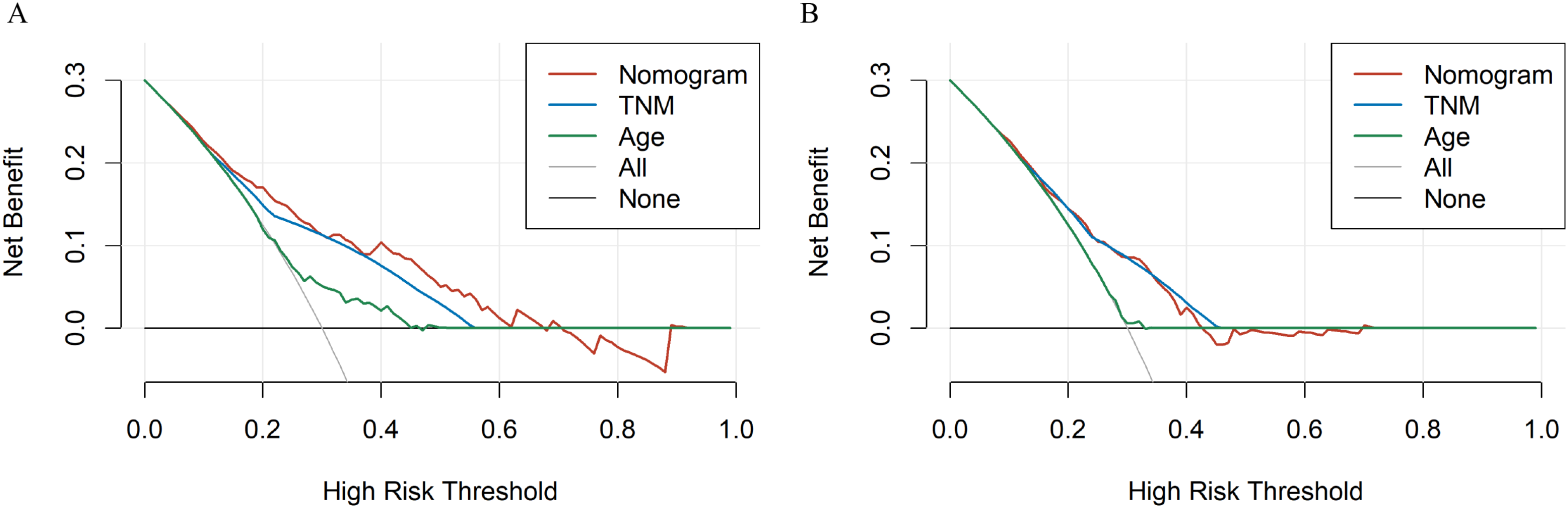
The DCA curves of the nomogram compared with prognosis based on TNM staging. *X*-axis represents high risk threshold and *Y*-axis represents net benefit. (A) DCA curve of the nomogram predicting OS compared with prognosis based on TNM staging. (B) DCA curve of the nomogram predicting CSS compared with prognosis based on TNM staging.

To evaluate accuracy of our nomograms, we analyzed ROC curves. The ROC curves indicated sensitivity and specificity of a clinical evaluating model. The *Y*-axis meant sensitivity and the *X*-axis meant false positive rate. Because TNM was an acknowledged clinical prognostic factor and age was a risk factor related to ATC, we compared our nomograms with the accuracy of TNM and age evaluations. The ROC curves of 6-month, 1-year, 2-year nomograms for OS and CSS were all superior to TNM and age evaluations (Fig. 7). To assess the practicability of our nomograms, we analyzed DCA (Fig. 8). DCA represented net benefits of clinical decisions. The *Y*-axis meant net benefit and the *X*-axis meant high risk threshold. The horizontal gray line meant all true negative rate and diagonal gray line meant all true positive rate. DCA suggested that nomogram for OS was superior to TNM and age evaluations, while nomogram for CSS was similar to TNM evaluation but superior to age evaluations. Furthermore, our model exhibited better discrimination compared based on NRI and IDI. The NRI for the 6-, 12- and 24-month follow-up examinations were 0.593 (95% CI [0.393–0.781]), 0.589 (95% CI [0.353–0.786]) and 0.473 (95% CI [0.103–0.705]), respectively. Similarly, the IDI for the 6-, 12- and 24-month follow-up examinations were 0.105 (*P* < 0.001), 0.092 (*P* < 0.001) and 0.061 (*P* < 0.001), respectively. Both NRI and IDI indicated a superior predictive ability of our model compared to TNM staging system.

## Discussion

ATC is a rare and typically aggressive malignant tumor with a poor prognosis. Though ATC is lethal, there are many other causes patients could die from including complications of local or distant diseases. Therefore, it’s necessary to build prognostic predictive models designed for the individual patient. Among all the predictive tools, nomogram performs well in predicting survival for many cancers (Iasonos et al., 2008). Several reports haven shown that nomograms are scientific and precise enough to make themselves an alternative standard which can compare to the traditional TNM staging systems (Wang et al., 2006; Mariani et al., 2005). Thus, the aim of this study was to develop the OS and CSS nomograms to precisely predict the survival of patients with ATC.

In our study, females and Whites are predominant risk factors with a female to male ratio of 1.67:1 and Whites to non-Whites ratio of 4.33:1, which were consistent with previous reports (Kebebew et al., 2005). Additionally, above half of the samples exhibited distant metastasis, similar to what has been reported before (Glaser et al., 2016). As for the year of diagnosis, we could see an increasing trend year by year although it has been reported that there was no obvious change in the incidence of ATC (Davies & Welch, 2006), even some data showed that the relative incidence of ATC has gradually decreased over time (Lee et al., 2016). This interesting trend might be attributable to increased use of ultrasonography and popularity of physical examination in recent years, reduction of incidence of goiter which was highly relevant to ATC via increasing in dietary iodine content.

Several studies have identified numerous factors including age, gender, clinical manifestation, tumor size, distant metastasis, leukocytosis, and acute symptoms as prognostic factors for ATC (Pierie et al., 2002; Lee et al., 2016). In our study, besides the factors mentioned above, we investigated several uncommon indicators such as year of diagnosis, historic stage, treatment modality (surgery and radiotherapy). For each risk factor, we used multivariate analysis, survival analysis, Cox regression analysis and competing risk analysis to obtain accurate conclusions. We found that historic stage was the most important prognostic factors among all the indicators as the risk of mortality for patients with distant metastasis was 2.285 times that of patients with localized primary tumors, which was parallel to previous studies (Kwon et al., 2016; Ito et al., 2012; Landa et al., 2016). Furthermore, the prognosis of people older than 65 was worse than that of people younger than 65 in our nomogram which was consistent with previous reports (Roche et al., 2018; Molinaro et al., 2017; Han et al., 2012; Sugitani et al., 2012).

The acute onset and rapid progress of ATC required a timely systematic and appropriate treatment plan for patients. And the appropriate treatment options recommended in American Thyroid Association (ATA) guidelines published in 2012 included surgery, radiotherapy and chemotherapy and the final therapy chosen for each patient should be based on their own conditions (Smallridge et al., 2012). Our results also showed a better prognosis in patients undertaking complete thyroid surgical resections compared with those patients who didn’t undergo surgery. What’s more, radiotherapy in ATC patients was associated with an improved OS and CSS in our study. It was consistent with what Minoru Kihara has found in his study that the prognosis of the patients with ATC depended on whether complete resection could be achieved (Kihara et al., 2004). Kebebew et al. (2005) showed in their study that combined surgical resection with external beam radiation treatment were associated with a better survival in patients with regional and distant disease. Another study conducted by Demeter JG et al. also reported that both operation and radiotherapy in ATC patients improved survival (Wachter et al., 2020). However, our data didn’t include information about chemotherapy, so it was hard to tell how chemotherapy effected prognosis of ATC patients. In other researches, it has been reported that patients who received radiotherapy and chemotherapy had a significant advantaged survival (Lin et al., 2019).

Although it has been reported that small tumors indicated better prognosis (Sugitani et al., 2012), we found that tumor size was not associated with prognosis of ATC patients. The interesting contradiction was attributable to specific tumor size. Tumor sizes less than or equal to 5–7 cm were favorable prognostic indicators of ATC. However, we studied tumor sizes less than 4 cm. As for gender, some studies reported no effects from gender on survival (Haigh et al., 2001; Sugitani et al., 2001; Kihara et al., 2004), some studies reported that gender was an independent prognostic factor (Roche et al., 2018). In the current study, we discovered that gender was not a prognostic factor in CSS and OS. A reasonable explanation for this was that many other causes could result in the deaths of ATC patients such as complications of local or distant diseases which counteracted the role of sex (Haddad et al., 2015). In addition, race and year of diagnosis had no effects on prognosis of ATC patients.

Nomogram is a user-friendly personalized prognostic tool that is useful for counseling patients, assisting the therapeutic decision-making process. To the best of our knowledge, our study was the first nomogram to estimate the OS and CSS of patients with ATC. Our model was reliable because of good C-index, calibration curves and ROC curves of internal validation and external validations which can support the accuracy of our model. Besides, DCA indicated that our nomograms had good practicability.

However, we had to admit that there were some limitations in our study. First of all, the cases included in our study was not big enough (1,404). What’s more, because the SEER database didn’t contain information regarding other thyroid diseases, we couldn’t evaluate the effects of these factors. Unfortunately, these factors played important roles in prognosis of ATC patients. For example, most of ATC patients had a history of goiter which was thought to be a risk factor for survival (Molinaro et al., 2017). Finally, the unavoidable selection bias did exist in all retrospective studies. We hoped that more future studies or research could be conducted to confirm our findings.

In summary, we identified age, surgery, radiotherapy, historic stage as prognostic predictor for the OS and CSS of patients with ATC. We also developed and validated prognostic nomogram for ATC, which we recommend using in patients with ATC to assist predicting the survival of the patients.

## Supplemental Information

10.7717/peerj.9173/supp-1 Supplemental Information 1 Spline plots for examination of non-linear relationships between age at diagnosis and survival months. *X*-axis represents age at diagnosis and *Y*-axis represents survival months.

## Abbreviations

ATC: anaplastic thyroid carcinoma
CSS: cancer specific survival
HR: hazard ratio
ICD-O: International Classification of Diseases for Oncology
OS: overall survival
SEER: Surveillance Epidemiology and End Results
ROC: receiver operating characteristic
DCA: decision curve analysis
NRI: net reclassification index
IDI: integrated discrimination index

## Additional Information and Declarations

### Data Availability

The following information was supplied regarding data availability: The data was downloaded from SEER STAT and is available at GitHub: https://github.com/Weiwei-Gui/SEER.git.

## References

[ref-1] Araque KA, Gubbi S, Klubo-Gwiezdzinska J (2020). Updates on the management of thyroid cancer. Hormone and Metabolic Research.

[ref-2] Davies L, Welch HG (2006). Increasing incidence of thyroid cancer in the United States, 1973–2002. JAMA.

[ref-3] Frank E (2019). http://cran.r-project.org/package=rms.

[ref-4] Gilliland FD, Hunt WC, Morris DM, Key CR (1997). Prognostic factors for thyroid carcinoma: a population-based study of 15,698 cases from the Surveillance, Epidemiology and End Results (SEER) program 1973–1991. Cancer.

[ref-5] Giuffrida D, Gharib H (2000). Anaplastic thyroid carcinoma: current diagnosis and treatment. Annals of Oncology.

[ref-6] Glaser SM, Mandish SF, Gill BS, Balasubramani GK, Clump DA, Beriwal S (2016). Anaplastic thyroid cancer: prognostic factors, patterns of care, and overall survival. Head & Neck.

[ref-7] Haddad RI, Lydiatt WM, Ball DW, Busaidy NL, Byrd D, Callender G, Dickson P, Duh QY, Ehya H, Haymart M, Hoh C, Hunt JP, Iagaru A, Kandeel F, Kopp P, Lamonica DM, McCaffrey JC, Moley JF, Parks L, Raeburn CD, Ridge JA, Ringel MD, Scheri RP, Shah JP, Smallridge RC, Sturgeon C, Wang TN, Wirth LJ, Hoffmann KG, Hughes M (2015). Anaplastic thyroid carcinoma, version 2.2015. Journal of the National Comprehensive Cancer Network.

[ref-8] Haigh PI, Ituarte PH, Wu HS, Treseler PA, Posner MD, Quivey JM, Duh QY, Clark OH (2001). Completely resected anaplastic thyroid carcinoma combined with adjuvant chemotherapy and irradiation is associated with prolonged survival. Cancer.

[ref-9] Han JM, Kim WB, Kim TY, Ryu JS, Gong G, Hong SJ, Kim JH, Oh YL, Jang HW, Kim SW, Chung JH, Shong YK (2012). Time trend in tumour size and characteristics of anaplastic thyroid carcinoma. Clinical Endocrinology.

[ref-10] Iasonos A, Schrag D, Raj GV, Panageas KS (2008). How to build and interpret a nomogram for cancer prognosis. Journal of Clinical Oncology.

[ref-11] Ito K, Hanamura T, Murayama K, Okada T, Watanabe T, Harada M, Ito T, Koyama H, Kanai T, Maeno K, Mochizuki Y, Amano J (2012). Multimodality therapeutic outcomes in anaplastic thyroid carcinoma: improved survival in subgroups of patients with localized primary tumors. Head & Neck.

[ref-12] Kebebew E, Greenspan FS, Clark OH, Woeber KA, McMillan A (2005). Anaplastic thyroid carcinoma: treatment outcome and prognostic factors. Cancer.

[ref-13] Kihara M, Miyauchi A, Yamauchi A, Yokomise H (2004). Prognostic factors of anaplastic thyroid carcinoma. Surgery Today.

[ref-14] Kitamura Y, Shimizu K, Nagahama M, Sugino K, Ozaki O, Mimura T, Ito K, Ito K, Tanaka S (1999). Immediate causes of death in thyroid carcinoma: clinicopathological analysis of 161 fatal cases. Journal of Clinical Endocrinology & Metabolism.

[ref-15] Kwon J, Kim BH, Jung HW, Besic N, Sugitani I, Wu HG (2016). The prognostic impacts of postoperative radiotherapy in the patients with resected anaplastic thyroid carcinoma: a systematic review and meta-analysis. European Journal of Cancer.

[ref-16] Landa I, Ibrahimpasic T, Boucai L, Sinha R, Knauf JA, Shah RH, Dogan S, Ricarte-Filho JC, Krishnamoorthy GP, Xu B, Schultz N, Berger MF, Sander C, Taylor BS, Ghossein R, Ganly I, Fagin JA (2016). Genomic and transcriptomic hallmarks of poorly differentiated and anaplastic thyroid cancers. Journal of Clinical Investigation.

[ref-17] Lee DY, Won J-K, Lee S-H, Park DJ, Jung KC, Sung MW, Wu HG, Kim KH, Park YJ, Hah JH (2016). Changes of clinicopathologic characteristics and survival outcomes of anaplastic and poorly differentiated thyroid carcinoma. Thyroid.

[ref-18] Lim H, Devesa SS, Sosa JA, Check D, Kitahara CM (2017). Trends in thyroid cancer incidence and mortality in the United States, 1974–2013. JAMA.

[ref-19] Lin B, Ma H, Ma M, Zhang Z, Sun Z, Hsieh IY, Okenwa O, Guan H, Li J, Lv W (2019). The incidence and survival analysis for anaplastic thyroid cancer: a SEER database analysis. American Journal of Translational Research.

[ref-20] Mariani L, Miceli R, Kattan MW, Brennan MF, Colecchia M, Fiore M, Casali PG, Gronchi A (2005). Validation and adaptation of a nomogram for predicting the survival of patients with extremity soft tissue sarcoma using a three-grade system. Cancer.

[ref-21] Molinaro E, Romei C, Biagini A, Sabini E, Agate L, Mazzeo S, Materazzi G, Sellari-Franceschini S, Ribechini A, Torregrossa L, Basolo F, Vitti P, Elisei R (2017). Anaplastic thyroid carcinoma: from clinicopathology to genetics and advanced therapies. Nature Reviews Endocrinology.

[ref-22] National Cancer Institute (2015). Surveillance, epidemiology, and end results. http://www.seer.cancer.gov/.

[ref-23] Onoda N, Sugitani I, Ito KI, Suzuki A, Higashiyama T, Fukumori T, Suganuma N, Masudo K, Nakayama H, Uno A, Yane K, Yoshimoto S, Ebina A, Kawasaki Y, Maeda S, Iwadate M, Suzuki S (2020). Evaluation of the 8th edition TNM classification for anaplastic thyroid carcinoma. Cancers.

[ref-24] Orita Y, Sugitani I, Amemiya T, Fujimoto Y (2011). Prospective application of our novel prognostic index in the treatment of anaplastic thyroid carcinoma. Surgery.

[ref-25] Perros P, Boelaert K, Colley S, Evans C, Evans RM, Gerrard Ba G, Gilbert J, Harrison B, Johnson SJ, Giles TE, Moss L, Lewington V, Newbold K, Taylor J, Thakker RV, Watkinson J, Williams GR, British Thyroid Association (2014). Guidelines for the management of thyroid cancer. Clinical Endocrinology.

[ref-26] Pierie JP, Muzikansky A, Gaz RD, Faquin WC, Ott MJ (2002). The effect of surgery and radiotherapy on outcome of anaplastic thyroid carcinoma. Annals of Surgical Oncology.

[ref-27] Roche AM, Fedewa SA, Shi LL, Chen AY (2018). Treatment and survival vary by race/ethnicity in patients with anaplastic thyroid cancer. Cancer.

[ref-28] Rousson V, Zumbrunn T (2011). Decision curve analysis revisited: overall net benefit, relationships to ROC curve analysis, and application to case-control studies. BMC Medical Informatics and Decision Making.

[ref-29] Scrucca L, Santucci A, Aversa F (2007). Competing risk analysis using R: an easy guide for clinicians. Bone Marrow Transplantation.

[ref-30] Siegel RL, Miller KD, Jemal A (2016). Cancer statistics, 2016. A Cancer Journal for Clinicians.

[ref-31] Smallridge RC, Ain KB, Asa SL, Bible KC, Brierley JD, Burman KD, Kebebew E, Lee NY, Nikiforov YE, Rosenthal MS, Shah MH, Shaha AR, Tuttle RM, American Thyroid Association Anaplastic Thyroid Cancer Guidelines (2012). American thyroid association anaplastic thyroid cancer guidelines, 2012 American Thyroid Association guidelines for management of patients with anaplastic thyroid cancer. Thyroid.

[ref-32] Sugitani I, Kasai N, Fujimoto Y, Yanagisawa A (2001). Prognostic factors and therapeutic strategy for anaplastic carcinoma of the thyroid. World Journal of Surgery.

[ref-33] Sugitani I, Miyauchi A, Sugino K, Okamoto T, Yoshida A, Suzuki S (2012). Prognostic factors and treatment outcomes for anaplastic thyroid carcinoma: ATC research consortium of Japan cohort study of 677 patients. World Journal of Surgery.

[ref-34] Wachter S, Vorlander C, Schabram J, Mintziras I, Fulber I, Manoharan J, Holzer K, Bartsch DK, Maurer E (2020). Anaplastic thyroid carcinoma: changing trends of treatment strategies and associated overall survival. European Archives Oto-Rhino-laryngology.

[ref-35] Wang L, Hricak H, Kattan MW, Chen HN, Scardino PT, Kuroiwa K (2006). Prediction of organ-confined prostate cancer: incremental value of MR imaging and MR spectroscopic imaging to staging nomograms. Radiology.

[ref-36] Xiao Z, Shi Z, Hu L, Gao Y, Zhao J, Liu Y, Xu Q, Huang D (2019). A new nomogram from the SEER database for predicting the prognosis of gallbladder cancer patients after surgery. Annals of Translational Medicine.

[ref-37] Yang L, Shen W, Sakamoto N (2013). Population-based study evaluating and predicting the probability of death resulting from thyroid cancer and other causes among patients with thyroid cancer. Journal of Clinical Oncology.

[ref-38] Zhang J, Fan J, Yin R, Geng L, Zhu M, Shen W, Wang Y, Cheng Y, Li Z, Dai J, Jin G, Hu Z, Ma H, Xu L, Shen H (2019). A nomogram to predict overall survival of patients with early stage non-small cell lung cancer. Journal of Thoracic Disease.

[ref-39] Zhou Z-R, Wang W-W, Li Y, Jin K-R, Wang X-Y, Wang Z-W, Chen Y-S, Wang S-J, Hu J, Zhang H-N, Huang P, Zhao G-Z, Chen X-X, Li B, Zhang TS (2019). In-depth mining of clinical data: the construction of clinical prediction model with R. Annals of Translational Medicine.

[ref-40] Zhu F, Li Y, Guo Z, Cao L, Feng D, Zhang T, Zhu W, Gong J (2020). Nomogram to predict postoperative Intra-abdominal septic complications after bowel resection and primary anastomosis for Crohn’s disease. Diseases of the Colon and Rectum.

