# Supplementary figures and images for: Development and validation of a prognostic nomogram to predict overall survival and cancer-specific survival for patients with anaplastic thyroid carcinoma
Source: PeerJ. 2020 May 21;8:e9173. doi: 10.7717/peerj.9173 (PMC7246027; doi:10.7717/peerj.9173)

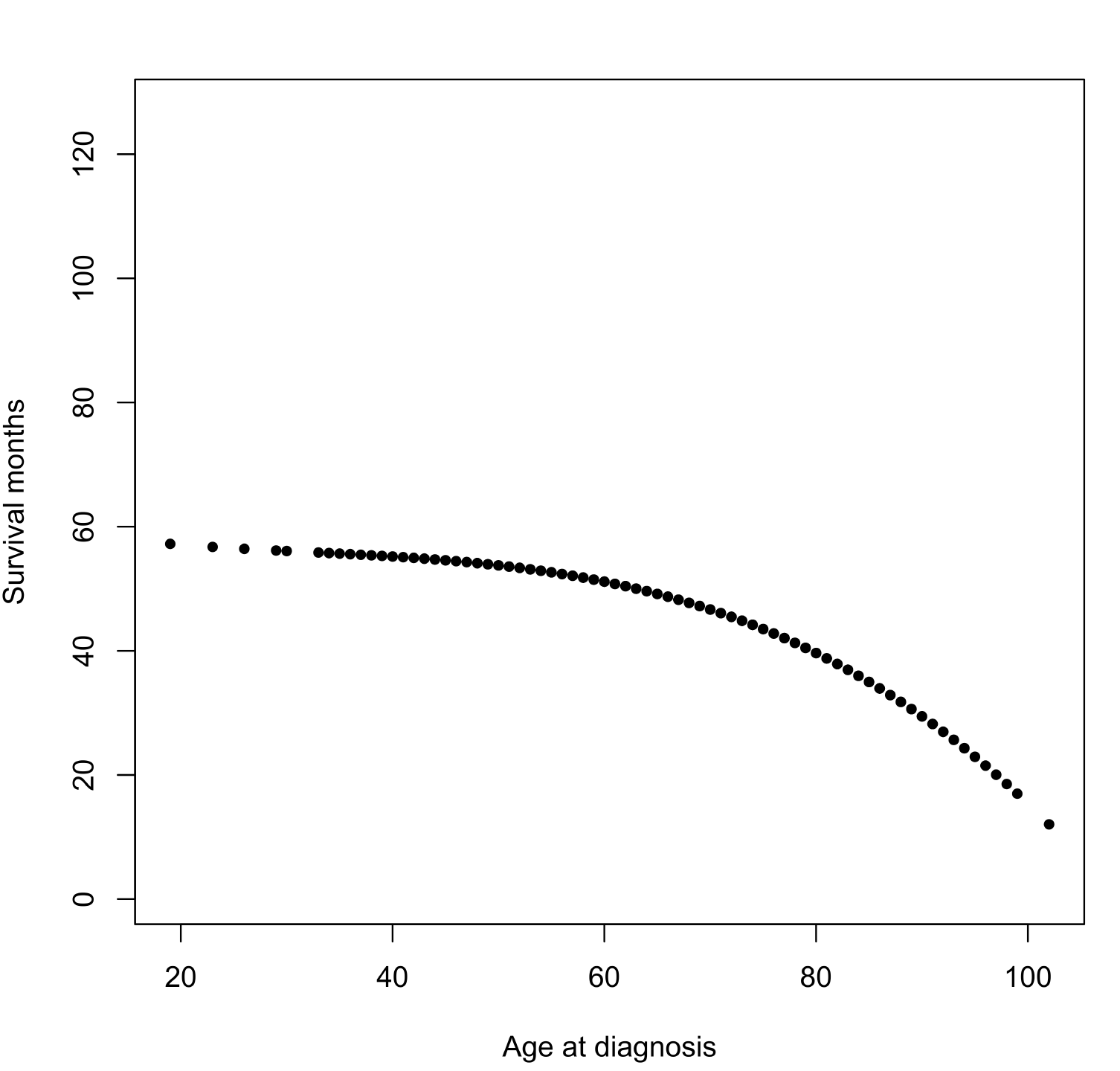

Supplement: Supplemental Information 1 — X-axis represents age at diagnosis and Y-axis represents survival months. [file peerj-08-9173-s001.png]
